# Prevalence and Mutation Patterns of HIV Drug Resistance from 2010 to 2011 among ART-Failure Individuals in the Yunnan Province, China

**DOI:** 10.1371/journal.pone.0072630

**Published:** 2013-08-29

**Authors:** Hanping Li, Min Zhong, Wei Guo, Daomin Zhuang, Lin Li, Yongjian Liu, Zuoyi Bao, Siyang Liu, Xiaolin Wang, Tianyi Li, Shaomin Yang, Jingyun Li

**Affiliations:** 1 Department of AIDS Research, State Key Laboratory of Pathogen and Biosecurity, Beijing Institute of Microbiology and Epidemiology, Beijing, China; 2 Yunnan Provincial Hospital of Infectious Disease, Kunming, China; Burnet Institute, Australia

## Abstract

**Background:**

Assessing the prevalence of HIV-1 drug-resistance and the mutation patterns associated with resistance in the geographical regions implementing free antiretroviral therapy (ART) in China is necessary for preventing the spread of resistant strains and designing the regimens for the subsequent therapies with limited resources.

**Methods:**

Plasma samples in different cities/prefectures were collected at Yunnan Provincial Hospital of Infectious Disease from January 2010 to December 2011. Genotyping of drug-resistant individuals was conducted using an in-house assay on plasma samples. Viral load, CD4 T cell counts and demographic data were obtained from medical records and an administered questionnaire.

**Results:**

A total of 609 pol sequences (515 ART-failure and 94 therapy-naïve individuals) derived from 664 samples were obtained. The prevalence of drug-resistance was 45.1% in the ART-failure individuals. Of these, 26.8% harbored HIV strains dually resistant to nucleoside reverse transcriptase inhibitors and non-nucleoside reverse transcriptase inhibitors, and 14.8% harbored HIV strains resistant to only one drug category. Mutations such as M184V/I, K103N, V106A, Y181C and G190A were common among the ART-failure individuals, and the frequencies of M184V/I, K103N and V106A were 28.2%, 19.2%, and 22.1%, respectively. The percentages of individuals exhibiting intermediate or high-level resistance to 3TC, FTC, EFV and NVP drugs were 28.4%, 28.2%, 37.3%, and 37.5%, respectively. Factors such as ethnicity, transmission route, CD4 counts, viral load and the duration of ART were significantly correlated with development of drug resistance in the ART-failure individuals.

**Conclusions:**

The high prevalence of HIV drug-resistance observed among the ART-failure individuals from 2010 to 2011 in Yunnan province should be of increasing concern in regions where the implementation of ART is widespread. Education about the risk factors associated with HIV drug resistance is important for preventing and controlling the spread of HIV drug-resistant strains.

## Background

It is estimated that at least 740,000 people live with HIV in China, and this epidemic is characterized by geographical disparities as well as a higher prevalence among certain sub-groups of the population [1]. Since 2009, the transmission route of sexual contact has overtaken intravenous drug use (IDU) and become more prevalent [2], with sexual transmission accounting for 59.0% (44.3% heterosexual, 14.7% homosexual) of the living AIDS/HIV individuals. In an attempt to prevent and control the spread of AIDS, the Chinese government implemented the National Free ART Program in 2003 and expanded the program in 2004 [3]. This program significantly contributed to a dramatic reduction in mortality among individuals receiving ART. However, this success has turned out to be a ‘double-edged sword’ in China as drug-resistant strains of HIV quickly emerged under the selective pressure of antiviral drugs among ART individuals [4]–[6], likely due to poor adherence in some ART implementation regions. This might directly result in lower availability of drug substitution options in future ART iterations in China as there are only nine free antiretroviral drugs (seven first-line drugs and two second-line drugs) available from the initiation of free ART to the present time. Initially, the regimen of the first-line treatment consisted of zidovudine (AZT or ZDV), stavudine (d4T) or didanosine (ddI) in combination with nevirapine (NVP) or efavirenz (EFV). With comprehensive evaluation of free antiretroviral drugs and regimens, ddI was replaced with lamivudine (3TC) due to its poor virological response and drug-resistance in 2005. In 2008, the second-line treatment became available through the national program [7], with boosted lopinavir (LPV/r) provided in combination with either d4T, AZT, 3TC, or ddI. In 2010, tenofovir (TDF) in combination with 3TC became available as the second-line treatment in combination with boosted LPV/r [8].

Yunnan, a southwestern province of China that shares a border with the well-known heroin-producing region of Myanmar (Burma), was once considered the epicenter of China [9]. In recent years, the flourishing commercial sexual trade and drug exchange at these border regions promoted the spread of HIV among sub-groups of sex workers and intravenous drug users (IDUs) so widely that the HIV epidemic was placed at the forefront of national issues [10]–[11]. To effectively prevent the spread of HIV and control the progression of AIDS in already-infected individuals, assessing the prevalence and mutation patterns of HIV-1 drug resistance in regions that have implemented the free ART is vital for the future. This study aimed to determine the prevalence and mutation patterns of HIV-1 drug-resistance among the ART-failure individuals from 2010 to 2011 in the Yunnan province and evaluate the risk factors that are associated with the development of HIV drug-resistance strains.

## Results

### Individual Characteristics

As of 2011, 13,736 individuals have been accepted to the free ART program in the Yunnan province, and the viral load (VL) had been determined at least once annually to evaluate the efficacy of ART. From 2010 to 2011, 1,066 individuals whose VL was more than 1000 copies/mL were found among the 13,736 individuals. A total of 503 plasma specimens did not undergo the genotyping resistance test among the 1066 individuals because of inadequate plasma volume (≤500 µL) and the duration of ART being less than 12 months. Genotyping resistance test was carried out on 664 plasma specimens, including 101 therapy-naïve control and 563 ART-failure individuals. After the genotyping test was performed using an in-house method and the raw sequences were assembled, aligned and edited, 609 pol sequences (515 ART-failure and 94 therapy-naïve individuals) were obtained and the amplification of 55 specimens was negative, achieving a positive ratio of 91.7% (609/664).

**Figure 1:**
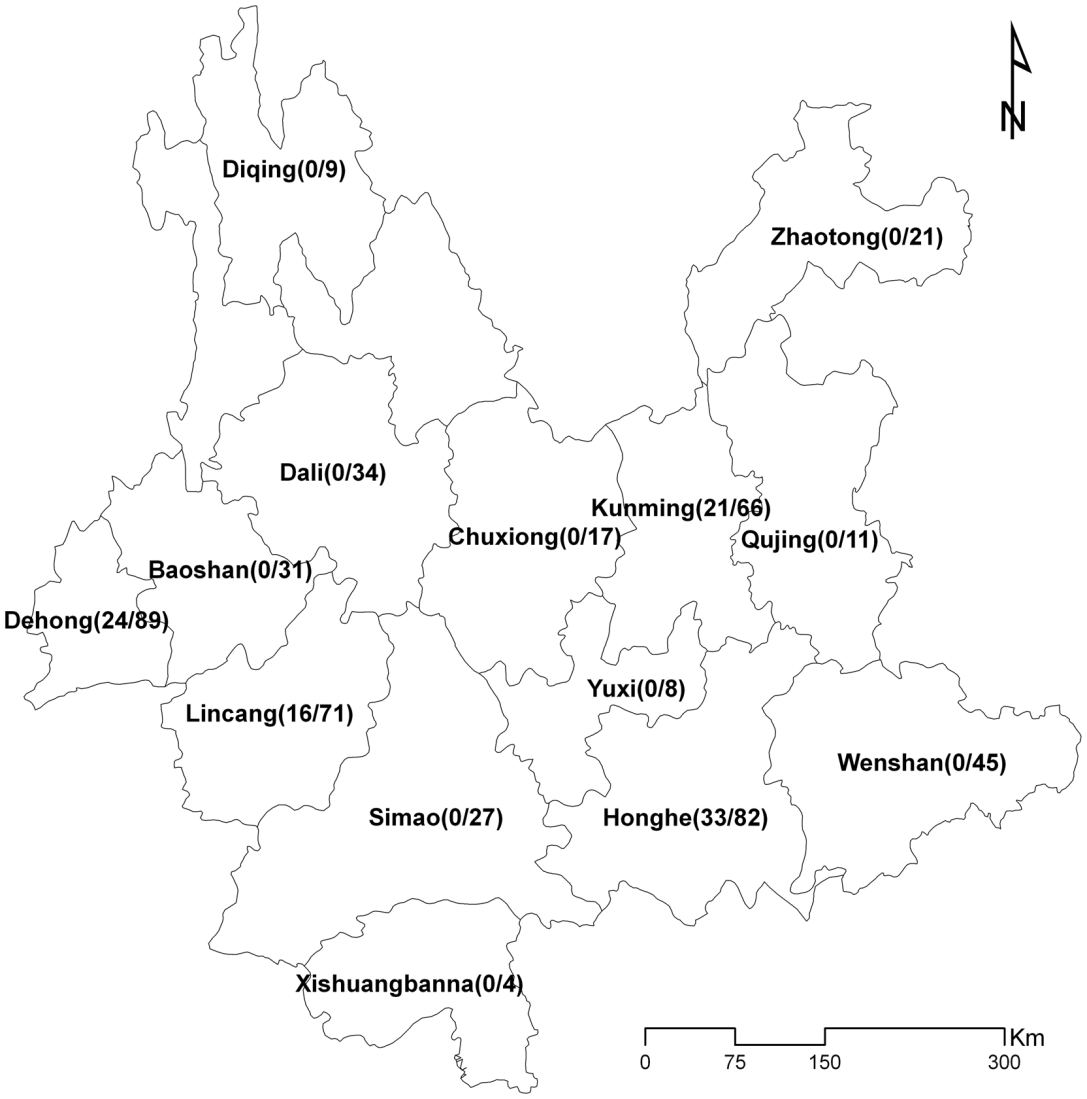
Geographical distribution of specimens collected in this study. Note: the numbers to the right and left of the “/”represent the therapy-naïve and the ART individuals, respectively.

According to self-reported answers to a questionnaire, the following individual characteristics were found. Individuals in the study originated from nearly all regions of the Yunnan province, including Kunming City, Lincang City, Honghe Prefecture, and Dehong Prefecture. Each region was represented by over 10% of the specimens (12.8%, 13.8%, 15.9%, and 17.3%, respectively; Figure 1). In terms of ethnicity, 71.6% were of the Han ethnicity and 28.4% were Dai origin or other minorities. The majority of individuals were male (54.3% therapy-naïve and 61.2% ART-failure individuals), and the median age was 39.3 years and 36.2 years in the therapy-naïve and ART-failure individuals, respectively (Table 1).

**Table 1:** Demographic characteristics of individuals in the study.

Characteristic	Individuals Number (%)		
	Naïve (n = 94)	ART (n = 515)	Cumulative (n = 609)
**Age, median year s(IQR** ^a^ **)**	39.3 (26–57)	36.2 (4–74)	38.5 (4–74)
**Gender**			
Male	51 (54.3)	315 (61.2)	366 (60.1)
Female	43 (45.7)	200 (38.8)	243 (39.9)
Median CD4+ T cell count, cells/µL (IQR)*	157 (11–317)	193 (2–1140)	177 (2–1140)
**Median VL, RNA(lgcopies/ml) (IQR)**	4.5 (2.5–6.2)	4.3 (3.0–5.9)	4.4 (2.5–6.2)
**HIV subtypes/CRFs**			
CRF07_BC	18 (19.2)	91 (17.7)	109 (17.9)
CRF08_BC	40 (42.6)	276 (53.6)	316 (51.9)
B	1 (1.1)	48 (9.3)	49 (8.1)
CRF01_AE	26 (27.7)	91 (17.7)	117 (19.2)
Unknown subtype/CRF	9 (9.6)	9 (1.8)	18 (3.0)
**Risk of HIV infection**			
Heterosexual*	73 (77.7)	256 (49.7)	329 (54.0)
Homosexual	11 (11.7)	5 (1.0)	16 (2.6)
IDU^b^	0	198 (38.5)	198 (32.5)
Blood	0	17 (3.3)	17 (2.8)
Others/Unknown	10 (10.6)	39 (7.6)	49 (8.1)
**Ethnicity**			
Han	40 (42.6)	396 (76.9)	406 (66.7)
Dai	18 (19.2)	27 (5.2)	45 (7.4)
Jingpo	13 (13.8)	24 (4.7)	37 (6.1)
Wa	5 (5.3)	17 (3.3)	22 (3.6)
Hui	7 (7.5)	12 (2.3)	19 (3.1)
Yi	2 (2.1)	11 (2.1)	13 (2.1)
Hani	1 (1.1)	8 (1.6)	9 (1.5)
Bai	2 (2.1)	4 (0.8)	6 (1.0)
Zhuang	1 (1.1)	3 (0.6)	4 (0.7)
Miao	1 (1.1)	2 (0.4)	3 (0.5)
Lahu	1 (1.1)	2 (0.4)	3 (0.5)
Weiwuer	1 (1.1)	1 (0.2)	2 (0.3)
Naxi	0 (0.0)	1 (0.2)	1 (0.2)
Unknown	2 (2.1)	6 (1.2)	8 (1.3)

Note: ^a^IQR, interquartile range;

b IDU, intravenous drug use; the proportion of FSWs was 86.8% (211/243) in the female group.

Comparing HIV-related characteristics between the two groups, the CD4+ T cell counts (χ^2^ = 2.42, *P*>0.05) and VL (χ^2^ = 1.23, *P*>0.05) were not statistical different between the two groups. The prevalent subtypes in this study were similar to previous reports (CRF08_BC 51.0%, CRF07_BC 24.5% and CRF01_AE 20.4%) [12]–[14], with subtype CRF08_BC being more prevalent in the population than subtypes CRF01_AE and CRF07_BC (51.9% vs. 19.2% and 17.9%, respectively). Consistent with the previously reported changes in the most common transmission route of the AIDS epidemic, we confirmed that sexual contact was the main transmission route in the study population at 56.7% (345/609; 54.0% heterosexual and 2.6% homosexual), followed by the IDU route (32.5%). In addition, the majority of female individuals were female sex workers (FSWs) at 86.8% (211/243).

**Figure 2:**
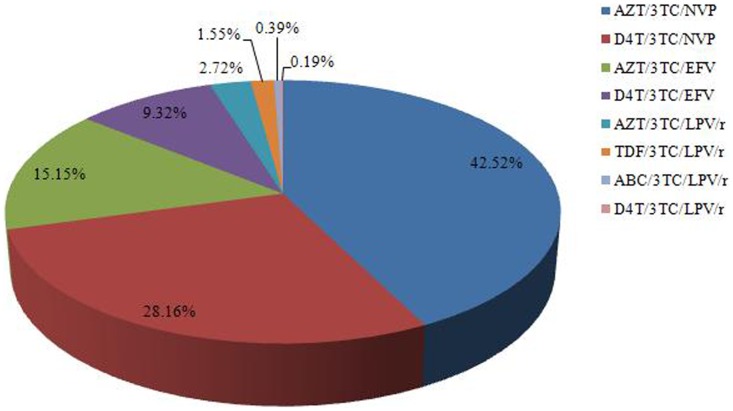
The regimens of ART individuals in the study.

Among all therapy regimens in ART-failure individuals (Figure 2), the AZT/3TC/NVP regimen was the most common at 42.5%. The proportion of individuals treated with d4T/3TC/NVP, AZT/3TC/EFV and d4T/3TC/EFV was 28.2%, 15.2%, and 9.3%, respectively. With the implementation of second-line antiviral drugs in China in recent years, 4.9% of the individuals accepted free protease inhibitor LPV/r therapy.

### Prevalence of HIV Drug Resistance (HIVDR) in ART-failure Individuals

**Figure 3:**
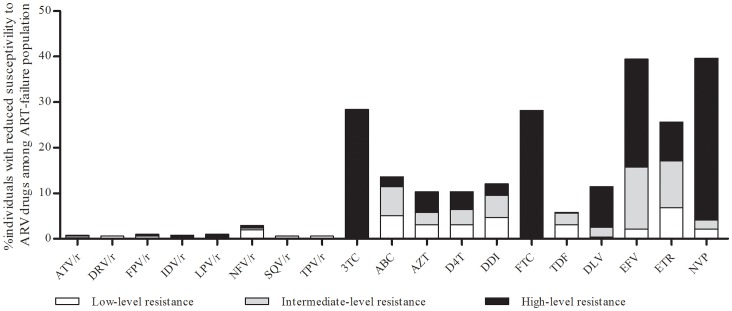
Antiretroviral drug resistance levels among the ART-failure individuals in the Yunnan province in 2010–2011. Note: DRV/r, darunavir with ritonavir; LPV/r, lopinavir with ritonavir; TPV/r, tipranavir with ritonavir; ABC, abacavir; FTC, emtricitabine; ETR, etravirine; DLV, delavirdin.

Next, we evaluated the presence of mutations in the HIV pol gene by comparing the sequences against the HIVDR Database from Stanford University to find the known mutations related with drug resistance. The prevalence of drug resistance was 2.1% (2/94) in the control therapy-naïve individuals. One individual that was infected through homosexual contact, exhibited a high level of resistance to nelfinavir with ritonavir (NFV/r) with a D30N mutation and conferred intermediate resistance to all nucleoside reverse transcriptase inhibitors (NRTIs) with T69ins. The other individual infected through heterosexual contact, had mutations of L90M and A71V and exhibited low-level resistance to atazanavir with ritonavir (ATV/r) and fosamprenavir with ritonavir (FPV/r), intermediate resistance to indinavir with ritonavir (IDV/r) and saquinavir with ritonavir (SQV/r), and high resistance to NFV/r. The HIV resistant strains were likely introduced through their sexual partners as neither individual had a previous history of drug use. The frequencies of some mutations, including A71TV, T69S, and V179D were relatively high between the therapy-naive and the ART-failure individuals but the impact on susceptibility to antiviral drugs was low; therefore, these mutations were not described in detail in this study. In contrast to the low prevalence of drug resistance in the therapy-naïve individuals, 45.1% (232/515) of the ART-failure individuals exhibited at least one drug-resistant mutation. Of the 232 individuals (Table 2), six, six and four cases were resistant to all, protease inhibitors (PIs), and NRTIs, respectively. One of the 515 ART-failure cases exhibited dual resistance to PIs and NNRTIs. The majority of individuals were resistant to NNRTIs, 14.8% to NNRTIs alone and 26.8% were dual resistant to NRTIs and NNRTIs. The percentages of resistance to 3TC, FTC, EFV, ETR, and NVP drugs were 28.4%, 28.2%, 39.4%, 25.6% and 39.6% resistance, respectively (Figure 3). Resistance to the other five drugs (ABC, AZT, d4T, ddI, and DLV) was more than 10% each.

**Table 2:** The proportion of drug-resistance in ART-failure individuals according to drug classes.

Drug classes^a^	Cases	Proportion (%)
PIs	6	1.2
NRTIs	4	0.8
NNRTIs	76	14.8
Dual resistance to PIs and NRTIs	1	0.2
Dual resistance to PIs and NNRTIs	1	0.2
Dual resistance to NRTIs and NNRTIs	138	26.8
Multiple resistance to PI, NRTI and NNRTI	6	1.2
Susceptibility	283	55.0

Note: ^a^PIs, Protease inhibitors; NRTIs, Nucleoside reverse transcriptase inhibitors; NNRTIs, Non-nucleoside reverse transcriptase inhibitors; the drug classes were exclusive of each other in the study population.

The mutations associated with drug resistance present in ART-failure individuals are listed in Table 3. M184V/I was the most prevalent mutation associated with resistance to NRTI, with a frequency of 28.2%. As for the most prevalent mutations associated with NNRTI, the frequencies of K103N and V106A were approximately 20% (19.2% and 22.1%, respectively). With the widespread use of AZT and d4T, the resistance mutations associated with these drugs, including K70R, T215F/Y and K219Q/E, were 4.5%, 6.8%, and 5.1%, respectively.

**Table 3:** Prevalence of drug-resistance mutations between the therapy-naïve (n = 94) and ART-failure population (n = 515) in the Yunnan province in 2010–2011.

Protease inhibitor			Nucleoside reverse transcriptase inhibitor			Non-nucleoside reverse transcriptase inhibitor		
Mutations	Frequency (%)		Mutations	Frequency (%)		Mutations	Frequency (%)	
	Naïve (n = 94)	ART-failure (n = 515)		Naïve (n = 94)	ART-failure (n = 515)		Naïve (n = 94)	ART-failure (n = 515)
L10F	0.0	0.6	M41L	0.0	3.7	A98G	0.0	2.1
V11I	0.0	1.0	K65R	0.0	0.2	K101Q	0.0	1.4
L23I	0.0	0.2	D67TG	0.0	0.8	K101E	0.0	3.7
D30N	1.1	0.0	D67N	0.0	3.9	K103N	0.0	19.2
L33F	0.0	1.2	T69D	0.0	1.0	K103R	0.0	2.9
M46IL	0.0	0.8	T69ins	1.1	0.0	V106A	0.0	22.1
Q58E	0.0	1.0	K70R	0.0	4.5	V106M	0.0	2.9
L76V	0.0	0.4	L74I	0.0	0.2	V108I	0.0	2.9
V82A	0.0	0.4	V75TAM	0.0	1.6	E138GQ	0.0	1.0
V82F	0.0	0.2	F116Y	0.0	0.2	Y181V	0.0	0.6
I84V	0.0	0.2	Q151M	0.0	0.6	Y181C	0.0	11.8
N88D	0.0	0.2	M184VI	0.0	28.2	Y188L	0.0	0.8
L90M	1.1	0.0	L210W	0.0	1.2	Y188C	0.0	0.8
			T215Y	0.0	2.7	Y188F	0.0	0.2
			T215F	0.0	4.1	G190S	0.0	0.6
			K219Q/E	0.0	5.1	G190A	0.0	12.4
						P225H	0.0	2.9
						F227L	0.0	1.2
						M230L	0.0	1.9
						K238T	0.0	1.0

### Risk Factors for HIV-1 Drug Resistance

Eight potential factors were taken into account in univariate logistic regression, as listed in Table 4. Ethnicity, CD4 counts, VL, transmission route, and duration of ART significantly correlated with drug resistance (*P*<0.05). To determine the risk factors associated with HIV-1 drug resistance, multivariable logistic regression analysis was performed using stepwise selection to explore which potential factors associated with development of HIV drug-resistance strains. We identified the following five potential factors that significantly correlated with development of drug resistance in the ART-failure individual: Ethnicity (others vs. Han: OR 1.6; 95% CI 1.0–2.5; *P* = 0.04); transmission route (drug injection vs. sexual contact transmission: OR 0.6; 95% CI 0.4–0.9; *P* = 0.02), (transfusion or other vs. sexual contact: OR 2.0; 95% CI 1.0–4.0; *P*<0.01); CD4 counts (200–349 vs. <200: OR 0.5; 95% CI 0.4–0.8; *P*<0.01), (≥350 vs. <200: OR 0.3; 95% CI 0.1–1.0; *P* = 0.03 );VL(5,000–9,999 vs. <5,000–1,000: OR 0.5; 95% CI 0.3–1.0, *P* = 0.07),(≥10,000 vs. <5,000–1,000: OR 0.6; 95% CI:0.4–0.9; *P* = 0.04); and duration of ART (27–52 vs. ≤26: OR 3.0; 95% CI 2.0–4.5; *P*<0.01), (>52 vs. ≤26: OR 2.6; 95% CI 1.5–4.6; *P*<0.01).

**Table 4:** Factors associated with HIV drug resistance among the ART-failure individuals.

Factors	Resistance% (N)	Crude OR^b^ (95% CI)	*P-value*	Adjusted OR(95% CI)	*P-value*
Total	45.1 (232)				
Gender					
Male	43.2 (136)	1.00	0.27		
Female	48.0 (96)	1.2 (0.9, 1.8)			
Ethnics					
Han	42.2 (167)	1.00	0.01	1.0	0.04
Others	54.6 (65)	1.7 (1.2, 2.6)		1.6 (1.0, 2.5)	
Age (years)			0.36		
<18	60.7 (17)	1.00			
18–44	45.0 (174)	0.5 (0.2, 1.2)			
≥45	44.2 (41)	0.5 (0.2, 1.2)			
Transmission route					
Sexual contact	47.1 (123)	1.0	0.02	1.0	<0.01
Drug injection	38.9 (77)	0.7 (0.5, 1.1)		0.6 (0.4, 0.9)	
Transfusion or other	57.1 (32)	1.7 (0.9, 3.0)		2.0 (1.0, 4.0)	
CD4 counts					
<200	53.5 (139)	1.0	<0.01	1.0	<0.01
200–349	37.9 (88)	0.5 (0.4, 0.8)		0.5 (0.4, 0.8)	
≥350	35.3 (5)	0.5 (0.2, 1.3)		0.3 (0.1, 1.0)	
VL					
<5,000–1,000	57.5 (64)	1.0	0.02	1.0	0.04
5,000–9,999	44.9 (134)	0.6 (0.4, 0.9)		0.5 (0.3, 1.0)	
≥10,000	34.7 (34)	0.4 (0.2, 0.7)		0.6 (0.4, 0.9)	
ART regimens					
AZT/3TC/NVP	44.8 (98)	1.0	0.08		
d4T/3TC/NVP	54.6 (78)	1.4 (0.9, 2.2)			
AZT/3TC/EFV	38.5 (30)	0.8 (0.5, 1.3)			
d4T/3TC/EFV	51.2 (21)	0.9 (0.5, 1.8)			
Others^a^	14.7(5)	0.5 (0.2, 1.2)			
Duration of ART(months)					
≤26	31.7 (68)	1.0	<0.01	1.0	<0.01
27–52	56.9 (123)	2.9 (1.9, 4.2)		3.0 (2.0, 4.5)	
>52	53.9 (41)	2.5 (1.5, 4.3)		2.6 (1.5, 4.6)	

Note: ^a^AZT/3TC/LPV/r, TDF/3TC/LPV/r, ABC/3TC/LPV/r, and d4T/3TC/LPV/r were included.

b OR, Odds Ratio.

## Discussion

As of 2009, a change occurred in the AIDS epidemic in China as sexual contact became the leading transmission route over IDU, exacerbating the difficulty of AIDS prevention due to the migratory nature of sex workers and their multiple sexual partners. With the implementation of free ART across the nation at approximately the same time, an increasing number of HIV/AIDS individuals infected through sexual contact accepted free ART [7]. This resulted in the spread of drug-resistance strains (developed after ART) from sex workers to the general population. In addition, an argument could be made that the spread of HIV drug-resistance would be higher among ART individuals because of a more ‘relaxed’ attitude towards HIV because they may think that their disease is under control.

Yunnan, the golden triangle border of drug trade due to its complicated geographical environment, has become the region with the highest prevalence of AIDS epidemic in China, where sexual contact has become the leading transmission route due to the influx of a large number of sex workers across the borders [10]–[11]. Therefore, the prevalence of AIDS rose with the flourishing of commercial sexual activity. Indeed, the characteristics of these migrant workers are as follows: young, mainly male, poorly educated, and frequent participants in the sex trade. Thus, we speculate that this population likely plays an important role in accelerating the spread of HIV/STIs due to the lack of using any protective measures during sexual intercourse [15]. Previously published studies provide further supporting evidence that the transmission probability from HIV-infected males to female sex workers was 0.2% (95% CI: 0.0014–0.0032) [16]. The prevalent ratios of HIV infection in female sex workers, men who have sex with men (MSM), and pregnant women rose drastically from 0.5% in 1995 to 4.0% in 2007, 4.0% in 2005 to 13.2% in 2007 and 0.2% in 1992 to 0.5% in 2007, respectively [9]. In addition, the study reported that 59.0% of their study population (44.3% heterosexual and 14.7% homosexual) acquired HIV through sexual contact as the main transmission route [4], suggesting that strategies to control and prevent the spread of HIV among individuals engaged in sexual service would be crucial for the subsequent work. It has also been demonstrated that the FSWs played an important role among female individuals because they constitute a high percentage of the female individuals.

With the comprehensive implementation of free ART initiated by the National Free Antiretroviral Treatment Manual across the nation, studies associated with resistance were conducted. The reports showed that 34.2% of former paid blood donor individuals in the Henan province [17] had at least one drug-resistance mutation to one antiviral drug, 23.7% were cross-resistant to NRTIs and NNRTIs after a 12-month duration and the prevalence of drug resistance in the ART individuals in a cross-section survey from 2004 to 2006 in the Hubei province [18]. In the present study, the prevalence of drug resistance was lower (2.1%) in the therapy-naïve than the ART-failure individuals, which was consistent with the results reported in the domestic study [19]–[20]. However, resistant strains were detected in 45.1% of the ART-failure individuals, implying that this high prevalence closely correlated with the widespread use of ART drugs. Additionally, our study demonstrated that the duration of ART positively correlated with increasing incidence of HIV-1 drug-resistant strains. i.e., the longer the duration of ART, the higher the probability of developing drug-resistance. Therefore, we conclude that the higher prevalence of HIV drug-resistant strains was found in individuals who exhibited lower adherence and longer duration of ART because it is difficult for individuals with AIDS to maintain strict adherence with painful side effects and long-term lifelong medication therapy.

As sexual contact was found to be the most common transmission route, sex workers would exacerbate the spread of HIV drug-resistant strains along with accelerating the spread of HIV in contrast with transmission of IDU. To prevent and control the spread of HIV among the IDUs, methadone maintenance treatment clinics were started in 2004 in China and generalized across the nation, which has been proven to be a cost-effective intervention for reducing HIV transmission and mortality due to better adherence among the IDUs [21]–[23] resulting in lower HIV drug resistance lower than in individuals infected through sexual contact. The possible cause for the high prevalence of HIV drug resistance among the individuals infected through transfusions was the longer duration of free-ART among these individuals than individuals infected through sexual contact and IDU as free ART was firstly initiated in these individuals [3].

The low CD4 T cell counts (below 200 cells/µL) showed a close correlation to the development of drug-resistant strains besides having a great impact on the risk of mortality [24]. This provided evidence for the monitoring of drug-resistance strains using CD4 T cell counts in the implementation of ART. An interesting phenomenon in this study was that the high risk of HIV drug resistance associated with VL occurred within 5,000–1,000 copies/ml. In defective viruses, the replication capacity of HIV drug resistant strains would be decreased during a course of viral DNA synthesis in contrast to the wild-type HIV virus, which has been proven by the published papers [25]–[28]; therefore, we deduced that the correlation about the high risk factor between the drug resistance of HIV and VL was negative.

China is a multiethnic nation and the inherent culture and religion are inherent within the minority ethnicities. The investigation conducted in southern provinces of China showed that drug abuse and use of condoms were regarded as “deviant behaviors” among the local minority because of their inherent culture and religion, which made prevention and control of HIV/AIDS more difficult due to the discrimination against drug abusers and rejecting usage of condom [29]–[33]. This predicament was also faced in other countries [34]. Different ethnic groups in Yunnan have their own national identities. Government and folk organizations have attempted to block the spread of HIV based on religious beliefs in some minority regions, but this has had little success because unprotected sexual behavior has been advocated among individuals with AIDS [35]. The result in this study showed that the occurrence of drug resistance significantly correlated with ethnicities, and in particular the ethnic minority in contrast to the Han majority. We assumed that the HIV resistance strains could quickly spread due to the higher risk of unprotected sexual behavior and the discrimination against drug abuse once they occur in minority individuals with AIDS.

The present study also has limitations. In particular, the fact that our study was based on a self-report questionnaire that included some questions about the characteristics of individuals and information on ART, which were completed verbally by individuals themselves. Judging the meaning of the data on adherence is difficult because we cannot confirm the accuracy. In addition, therapy-naïve samples were difficult to collect in this study due to the wide availability of free ART therapy. Thus, the therapy-naïve samples used in this study may not provide an appropriate control for all tested regions, and our drug resistance analysis might therefore deviate from actual numbers. A higher correlation between the minority ethnicities and the prevalence of HIV drug-resistance was found in this study. Genetic differences between the Han majority and the minority populations are likely and further studies are required on this issue.

In conclusion, the prevalence of HIV-1 drug-resistance in the ART-failure individuals of the Yunnan Province likely keeps them in a hyper-endemic state, especially for individuals that received free first-line ART drug therapy. Developing strategies to effectively optimize these regimens in local regions and promote adherence to long-term medication will be an arduous task, but will be important and of great significance to improve AIDS prevention and control.

## Materials and Methods

### Ethics Statement

Plasma samples were collected at the Yunnan Provincial Hospital of Infectious Disease. Written informed consent was obtained from every participant before blood sample donation. The study was revised and approved by the Ethics Committee of the Institutional Review Board of the Academy of Military Medical Sciences.

### Individuals

Between January 2010 and December 2011, a cross-sectional study was conducted between the naïve-therapy individuals and individuals who accepted the free ART for over 12 months. Self-reported demographic data were collected via a verbal questionnaire administered before sample donation. VL was measured with the NucliSENS EasyQ HIV-1 assay (Biomérieux, Lyon, France), and FACS analysis evaluating CD4 T cell counts were performed for each individual. ART-failure individuals were selected on the criteria that VL was more than 1000 copies/mL after 12 months of treatment. Then, HIV genotyping test was conducted on these selected individuals. The administration of ART, the delivery of antiviral drugs and the collection of plasma specimens were mainly depended on the local centers for disease control and prevention and the hospital for infectious disease. The evaluation of adherence was derived from the self-report questionnaire, which indicated missing doses of antiviral drugs during the treatment course. Most treatment naïve individuals recruited in this study accepted the free ART immediately after the baseline determination of theirs low CD4 counts (<200 cells/µl) and development of clinical symptoms.

### HIV Genotyping Drug Resistance Determination

A fragment of the HIV pol gene comprising the whole protease and 300 codons of the reverse transcriptase (RT) was bulk-sequenced from plasma HIV RNA using an in-house method developed in core laboratories that design tests for drug-resistant HIV strains in China. The test was conducted using two primer sets (Table S1). A two-step PCR amplification was performed to detect HIV-1 pol gene mutations. All samples were first tested using Primer Set I. RT-PCR was conducted using a one-step RNA PCR kit (TaKaRa Biotechnology Co. Ltd.). DR1–1 and DR1–2 (20 µM) were used as the forward and reverse primers, respectively. The PCR conditions were as follows: RNA was denatured at 65°C for 30 s, the reaction mixtures were added at 4°C, the samples were incubated at 50°C for 30 min and 94°C for 2 min, and the samples underwent 35 cycles of 94°C for 30 s, 55°C for 30 s and 72°C for 150 s. The second round was performed according to the following procedure in 50 µL reaction mixtures, each containing 25 µL of Premix Taq (Takara PerfectShot™ Ex Taq), 1 µL each of DR1–3 and DR1–4 primer (20 µM), 5 µL of the Primer Set I product and 18 µL ddH_2_O. The reactions were carried out for 35 cycles at 94°C for 30 s, 63°C for 63 s and 72°C for 150 s. The samples not detected by agarose gel electrophoresis were amplified using Primer Set II and the same reaction conditions as Primer Set I.

Genotyping drug resistance analyses of the mutations found in our study were carried out against protease inhibitors (PIs), NRTIs and NNRTIs using the Stanford HIV drug resistance database algorithm, and sequence quality was assessed using the WHO sequence quality-assessment tool. HIV subtype was performed using the Stanford calibrated population resistance tool. In addition, phylogenetic analysis and Simplot software, version 3.5.1, were used to further characterize unique recombinant forms.

### Data Analysis

All data were analyzed using SPSS for Windows Version 14.0 (SPSS, Chicago, IL, USA). Descriptive statistics were generated for each of the variables corresponding to specific questions in the survey. Chi-square tests were conducted to compare basic characteristics between the two groups. A multivariable logistic regression analysis model was used to identify factors associated with drug resistance. A stepwise approach was used for variable selection in multivariate regression model. Variables exhibiting a statistically significant association (*P*<0.05) with drug resistance in bivariate analyses were also evaluated. All statistical tests were two-sided with statistical significance defined as a value of *P*<0.05.

## Supporting Information

Table S1 **Primers for determining of HIV genotyping drug resistance.**
(DOC)
