# Supplementary material for: Prevalence and Mutation Patterns of HIV Drug Resistance from 2010 to 2011 among ART-Failure Individuals in the Yunnan Province, China
Source: PLoS One. 2013 Aug 29;8(8):e72630. doi: 10.1371/journal.pone.0072630 (PMC3757030; doi:10.1371/journal.pone.0072630)
Supplement: Table S1 — Primers for determining of HIV genotyping drug resistance. (DOC) [file pone.0072630.s001.doc]

**Table S1** Primers for determining of HIV genotyping drug resistance.

| Primer | Sequence (5′-3′) | Location (HXB2) |
| --- | --- | --- |
| *Set-I* |  |  |
| DR1-1 | TTGGAAATGTGGAAAGGAAGGAC | 2028→2050 (Sense) |
| DR1-2 | CTGTATTTCTGCTATTAAGTCTTTTGATGGG | 3539→3509 (Antisense) |
| DR1-3 | CAGAGCCAACAGCCCCACCA | 2147→2166 (Sense) |
| DR1-4 | CTGCCAGTTCTAGCTCTGCTTC | 3462→3441 (Antisense) |
| DR1-S1^a^ | GCCAACAGCCCCACCA | 2151→2166 (Sense) |
| DR1-S2 ^a^ | GGACCTACACCTGTCAAC | 2484→2501 (Sense) |
| DR1-S3 ^a^ | CCTAGTATAAACAATGAGACAC | 2946→2967 (Sense) |
| DR1-S4 ^a^ | GCTGGGTGTGGTATTCC | 3144→3128 (Antisense) |
| DR1-S5 ^a^ | GTTCTAGCTCTGCTTC | 3456→3441 (Antisense) |
| *Set-II* |  |  |
| DR2-1 | TGGAAATGTGGAAAAGAAGGAC | 2029→2050 (Sense) |
| DR2-2 | CTGTATTTCAGCTATCAAGTCTTTTGATGGG | 3539→3509 (Antisense) |
| DR2-3 | CAGAGCCAACAGCCCCACCA | 2147→2166 (Sense) |
| DR2-4 | CTGCCAATTCTAATTCTGCTTC | 3462→3441 (Antisense) |
| DR2-S1 ^a^ | CAGAGCCAACAGCCCCACCA | 2147→2166 (Sense) |
| DR2-S2 ^a^ | GGACCTACACCTGTCAAC | 2484→2501 (Sense) |
| DR2-S3 ^a^ | CCTAGTATAAACAATGAGACAC | 2946→2967 (Sense) |
| DR2-S4 ^a^ | GCTGGGTGTGGTATTCC | 3144→3128 (Antisense) |
| DR2-S5 ^a^ | CTGCCAATTCTAATTCTGCTTC | 3462→3441 (Antisense) |

Note: ^a^ Primers are used to the sequencing.
